# Mcl-1 Inhibition: Managing Malignancy in Multiple Myeloma

**DOI:** 10.3389/fphar.2021.699629

**Published:** 2021-07-19

**Authors:** Omar S. Al-Odat, Max von Suskil, Robert J. Chitren, Weam O. Elbezanti, Sandeep K. Srivastava, Tulin Budak-Alpddogan, Subash C. Jonnalagadda, Bharat B. Aggarwal, Manoj Pandey

**Affiliations:** ^1^ Department of Biomedical Sciences, Cooper Medical School of Rowan University, Camden, NJ, United States; ^2^ Department of Chemistry and Biochemistry, Rowan University, Glassboro, NJ, United States; ^3^ Department of Hematology, Cooper Health University, Camden, NJ, United States; ^4^ Department of Biosciences, Manipal University Jaipur, Jaipur, India; ^5^ Inflammation Research Center, San Diego, CA, United States

**Keywords:** multiple myeloma, drug resistant, Mcl-1, Bcl-2 homology 3 mimetics, apoptosis

## Abstract

Multiple myeloma (MM) is a plasma cells neoplasm. The overexpression of Bcl-2 family proteins, particularly myeloid cell leukemia 1 (Mcl-1), plays a critical role in the pathogenesis of MM. The overexpression of Mcl-1 is associated with drug resistance and overall poor prognosis of MM. Thus, inhibition of the Mcl-1 protein considered as a therapeutic strategy to kill the myeloma cells. Over the last decade, the development of selective Mcl-1 inhibitors has seen remarkable advancement. This review presents the critical role of Mcl-1 in the progression of MM, the most prominent BH3 mimetic and semi-BH3 mimetic that selectively inhibit Mcl-1, and could be used as single agent or combined with existing therapies.

## Introduction

**FIGURE 1:**
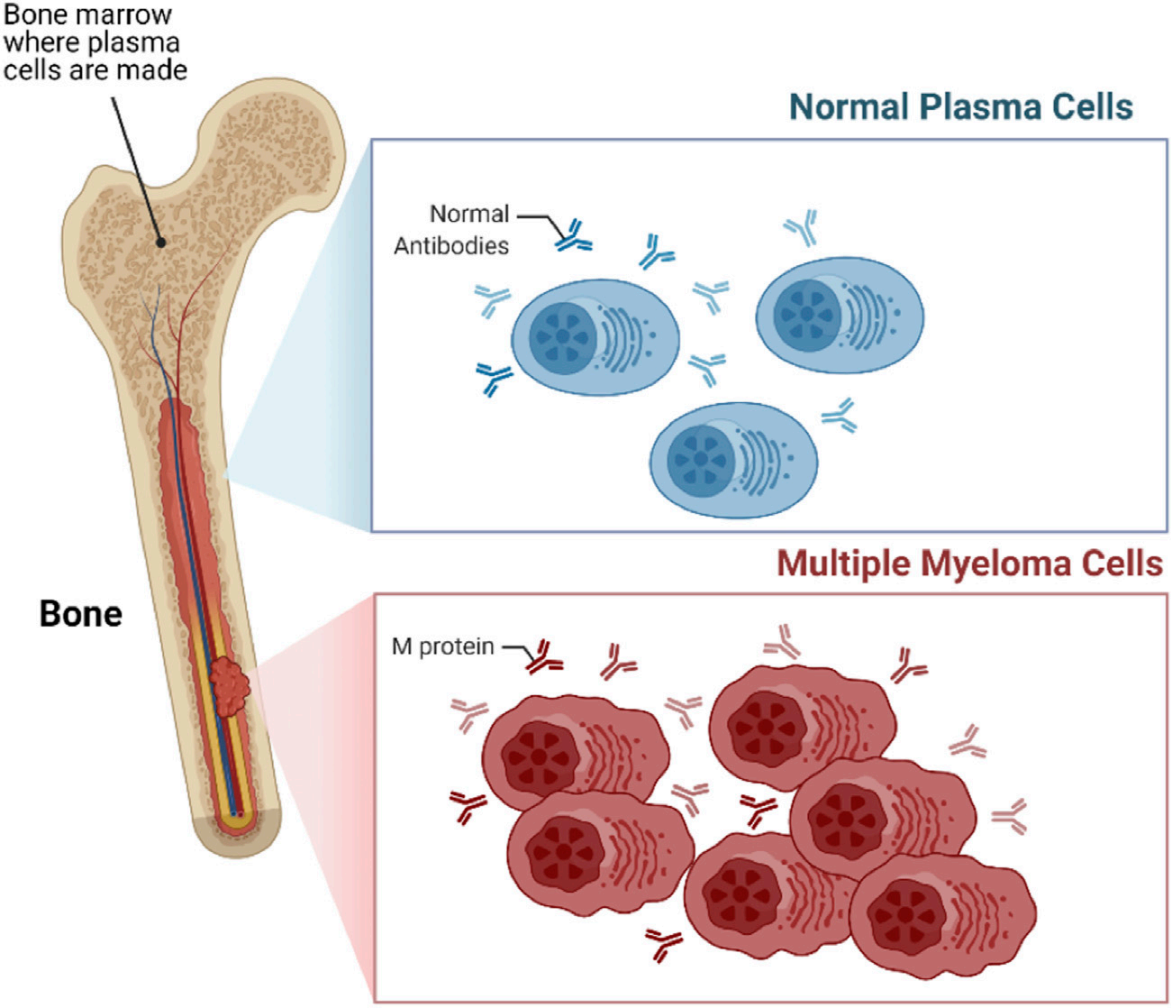
Multiple myeloma (MM). MM is a type of blood cancer that initiates from the bone marrow (BM), arising from the aberrant proliferation of plasma cells.

The innate and adaptive immune system comprises several different types of cells that elegantly work together to stave off infection and remove transformed or damaged cells. Lymphocytes including both T- and B-cells are among the most important cellular category within the immune system. The plasma cells are a type of unique B cells that reside in the bone marrow (BM) and secrete an antibody corresponding to the antigen. When these plasma cells begin proliferating out of control, they can build up within the BM and form numerous tumors across the body (Figure 1). This type of neoplasms is called Multiple Myeloma (MM) and considered the second most common hematologic malignancy, accounting around 12% of hematological malignancies (Kazandjian, 2016). MM is slightly more common among older men, with the median age of 65 years, and it is rarely diagnosed in younger people (Kumar et al., 2008; Gerecke et al., 2016; Naymagon and Abdul-Hay, 2016). Depending on the stage of disease, symptoms of MM begin with abnormalities in the bone and calcium homeostasis, low blood cell counts, renal insufficiency, and multiple infections. Because the symptoms are so generalized, MM is a challenging disease to diagnose. Furthermore, the protective role of the BM on the proliferating plasma cells make it even more challenging to treat.

In the last several decades the treatment options for MM have dramatically improved, unfortunately, the survival rate is marginal (Bergsagel and Kuehl, 2005). According to the American Cancer Society 2021 estimation, approximately 34,920 new MM cases will be diagnosed (19,320 men and 15,600 women), approximately 12,410 cancer deaths (6,840 men and 5,570 women) from MM alone in the United States (Siegel et al., 2021). Table 1 illustrates the common drugs that have been used to treat MM patients. Most therapeutic approaches to date for MM patients, especially in relapsed/refractory (R/R) cases have been based on combined formulations of available therapies. In spite of the efficacy and diversity of therapeutic approaches, drug resistance is a major challenge as MM continues to show high rates of relapse and quickly acquired resistance to therapies (Abdi et al., 2013). There are several unanswered questions regarding MM including: what are the causes of progression of MM from its precursor state? Why MM patients instigate to relapse? How MM clones resistant to drugs persist in the presence of effective therapies?

**TABLE 1:** Mechanism of action and Side effects of Common Therapy in MM.

Drug	Mechanism of action	Side effects	Ref
Melphalan	Chemotherapy drug	Bone marrow damage and chemotherapy side effects	Bergsagel et al. (1962)
Thalidomide (Thalomid)	Immunomodulating agent	Drowsiness, fatigue, constipation, and painful nerve damage as well as severe birth defects when taken during pregnancy	Singhal et al. (1999)
Bortezomib (Velcade)	Proteasome inhibitor	Vomiting, tiredness, diarrhea, constipation, decreased appetite, fever, lowered blood counts and nerve damage	Richardson et al. (2003)
Lenalidomide (Revlimid)	Small molecule analogue of thalidomide	Drowsiness, fatigue, constipation, and painful nerve damage as well as severe birth defects when taken during pregnancy	Hideshima et al. (2000), Rajkumar et al. (2005), Richardson et al. (2002)
Carfilzomib (Kyprolis)	Proteasome inhibitor	Tiredness, nausea, vomiting, diarrhea, shortness of breath, fever and low blood counts and occasionally more serious problems such as pneumonia, heart problems, and kidney or liver failure	Herndon et al. (2013)
Pomalidomide (Pomalyst)	Small molecule analogue of thalidomide	Same thalidomide side effects with a less risk of nerve damage side effect	Lacy et al. (2009)
Panobinostat (Farydak)	Oral Histone deacetylase (HDAC) inhibitor	Feeling tired, weakness, nausea, diarrhea vomiting, loss of appetite, fever, swelling in the arms or legs, and occasionally altered blood cell counts and blood electrolytes. Rare cases of internal bleeding, liver damage, and changes in heart rhythm which can sometimes be life threatening	Laubach et al. (2015)
Ixazomib (Ninlaro)	Oral proteasome inhibitor	Nausea, vomiting, diarrhea, constipation, swelling in the hands or feet, back pain, lowered blood platelet count and nerve damage	Muz et al. (2016)
Daratumumab (Darzalex)	Intravenous monoclonal antibody	Coughing, wheezing, trouble breathing, throat tightness, runny nose, nasal congestion, feeling dizzy or lightheaded, headache, rash, nausea, fatigue, back pain, fever, and lower blood cell counts	Lokhorst et al. (2015)
Elotuzumab (Empliciti)	Intravenous monoclonal antibody	Chills, feeling dizzy or lightheaded, wheezing, trouble breathing, cough, tightness in the throat, runny nose, nasal congestion, upper respiratory tract infections and pneumonia, rash, fatigue, loss of appetite, diarrhea, constipation, fever, and nerve damage	Lonial et al. (2015)
Selinexor (Xpovio)	Oral Nuclear export inhibitor of XPO1	Diarrhea, nausea, vomiting, loss of appetite, weight loss, low blood sodium levels susceptibility to infection, low platelet counts, and low white blood cell counts	Vogl et al. (2018)

## Mcl-1 Protein as a Potential Target for Multiple Myeloma

**FIGURE 2:**
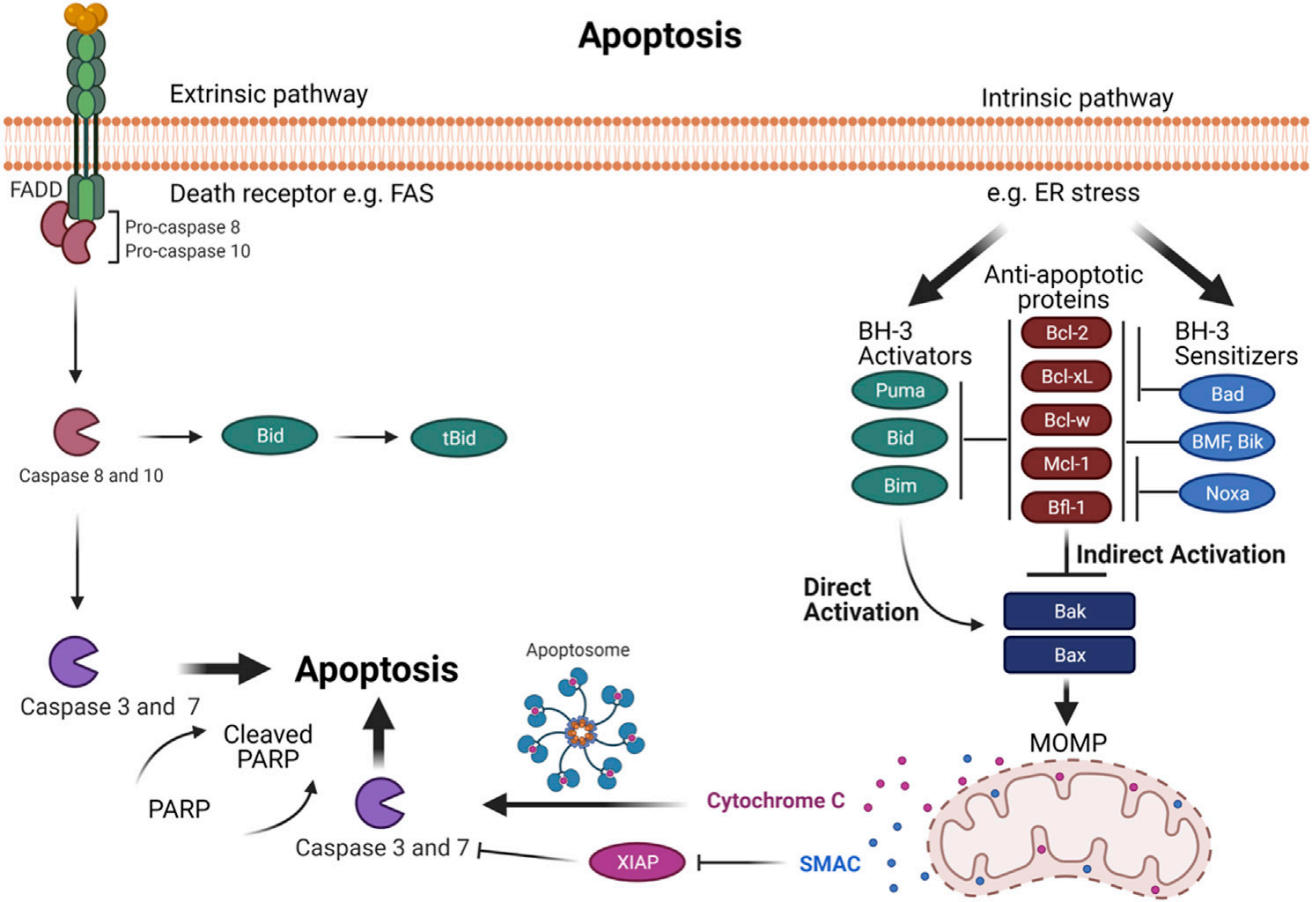
The programmed cell death- *via* intrinsic and extrinsic pathways in normal mammalian cells. Intrinsic and extrinsic pathways result in the activation of a family of protease enzymes called Caspase proteins. The intrinsic pathway is promoted by cellular stresses that modulate Bcl-2 family proteins and activates Bak and Bax. In the indirect activation, upregulation of BH3-only proteins will act as inhibitors of anti-apoptotic proteins by competing for their binding with Bax and Bak proteins, leading Bax and Bak to oligomerize. In the direct activation, upregulation of BH3 activators proteins directly activates Bax and Bak. This activation leads to mitochondrial outer membrane permeabilization (MOMP), subsequently Cytochrome C and SMAC proteins release into the cytosol, causing the downstream of Caspase activation that ends with apoptosis. The extrinsic pathway is promoted by death receptors activation. This leads to activation of initiator Caspases 8 and 10, which can regulate the downstream executioner Caspase such as Caspase 3 and 7 to drive full commitment to apoptosis. Moreover, Caspases 8 and 10 can activate Bid, which in turn activates Bak and Bax to induce MOMP which establishes the link between the extrinsic and intrinsic pathways.

Apoptosis is a vital procedure for regular development and maintaining the tissue homeostasis. Mammalian apoptosis occurs *via* one of two distinct pathways, either the intrinsic or extrinsic pathways (Figure 2). Both the intrinsic and extrinsic pathways end with the activation of a certain group of protease enzymes called Caspase proteins. The intrinsic pathway entails mitochondrial outer membrane permeabilization (MOMP) that regulates directly by interactions between B cell lymphoma 2 (Bcl-2) family proteins. The Bcl-2 family proteins are critical regulators of apoptosis. The members of this family proteins are divided into three groups according to function: anti-apoptotic proteins (Bcl-2, Mcl-1, Bcl-xL, Bcl-W, and Bfl-1); pro-apoptotic BH3-only proteins (Noxa, Puma, Bim, Bid, Bad, BMF, and Bik); and multi-domain pro-apoptotic proteins (Bax, Bak, and Bok). Intrinsic pathways like cytokine deprivation or DNA damage promote overexpression and activation of BH3-only proteins, which stimulate apoptosis in two different ways. First, the BH3-only proteins behave as inhibitors of anti-apoptotic proteins by competing for their binding with Bax and Bak proteins (Czabotar et al., 2014; Figure 2). This is accomplished *via* the amphipathic α-helix of the BH3 domain that contains four hydrophobic residues (h1-h4) that bind four hydrophobic pockets (P1−P4) within the anti-apoptotic proteins in their BH3 binding groove (Liang and Fesik, 1997; Czabotar et al., 2007; Stewart et al., 2010). For example, Noxa selectively inhibits Mcl-1 with high affinity binding thereby indirectly activating the Bax/Bak pathway (Smith et al., 2011; Kale et al., 2018). Simultaneously, BH3-only proteins can also result in the direct activation of multi-domain pro-apoptotic proteins Bax and Bak, which cause MOMP, leading to release of Cytochrome C and SMAC proteins into the cytosol resulting in downstream Caspase activation and ultimately activation of apoptosis (Dewson and Kluck, 2009).

The extrinsic pathway is promoted by death receptors activation. This leads to activation of initiator Caspases 8 and 10, which can directly induce the downstream executioner Caspase such as Caspase 3 and 7 to drive full commitment to apoptosis (Kaufmann et al., 2012). Moreover, Caspases 8 and Caspase 10 can activate Bid, which in turn activates Bak and Bax to induce MOMP, which is the connecting link between the extrinsic and intrinsic pathways (Kaufmann et al., 2012; Figure 2).

The mitochondrial membrane engages Mcl-1 with other Bcl-2 family partners for initiation of apoptosis. The interaction between the family members determine the outcome (Kale et al., 2018). Mcl-1 has a diverse localization within human cells. It is primarily found within the mitochondrial outer and inner membranes (Yang et al., 1995). However, studies have reported its localization in the nucleus and cytoplasm of polymorphonuclear leukocytes (PMNs) (Leuenroth et al., 2000). How different localization affects the function and its stability is not known.

The studies of Kozopas et al. first proved a high Mcl-1 expression in a differentiating human myeloid leukemia ML-1 cell line (Kozopas et al., 1993). Subsequently, it was shown to be expressed in several different cells as well. The MM cells exhibit imbalances in their anti-apoptotic proteins expression levels, especially Mcl-1 that leads to defects in the mitochondrial intrinsic pathway (Derenne et al., 2002; Zhang et al., 2002). In order to prevent apoptosis and allow continued cell growth, Mcl-1 forms a heterodimer protein-protein interaction with multi-domain pro-apoptotic proteins Bax and Bak (Sedlak et al., 1995; Willis and et al., 2005). Mcl-1 is known to be highly expressed in MM cells and plays a pivotal role in MM initiation, progression, and apoptosis resistance (Derenne et al., 2002; Zhang et al., 2002). Newly diagnosed cases of MM have continued to show increasing Mcl-1 protein expression, which predicts a higher relapse and poor patient survival rate (Wuillème-Toumi et al., 2005). Thus, Mcl-1 is an attractive therapeutic target for MM.

## Regulation of Mcl-1 Protein

**FIGURE 3:**
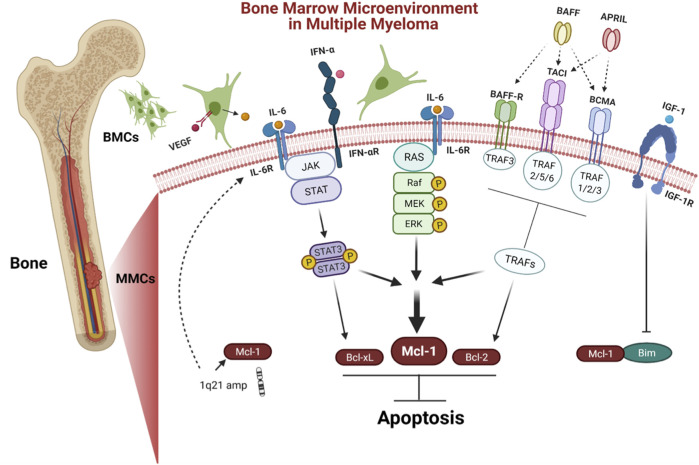
Bone marrow microenvironment (BMM) in MM. BMM facilitates the long term survival of MM. Stromal cells in BM regulate anti-apoptotic proteins by secreting a variety of signaling molecules including IL-6 and IFN-α that trigger JAK/STAT pathway, leading to the upregulation of Mcl-1, Bcl-xL, and VEGF. VEGF promotes IL-6 induction in neighboring BMCs. Furthermore, IL-6 induces survival of MM cells *via* Ras/MAPK pathway, which modulates the expression of Mcl-1 The tumor necrosis factor (TNF) family including BAFF and APRIL are other stimuli from the BMM that induce expression of both Mcl-1 and Bcl-2 *via* tumor necrosis factor receptor-associated factors (TRAFs) including BAFF-R, BCMA, and TACI. IGF-1 is another stimulus that acts by downregulating Bim, leading to release Mcl-1. MM cases have shown chromosomal amplification of 1q21 region, where the gene coding for Mcl-1 and IL-6R are located.

The interaction of myeloma cells to BM microenvironment (BMM) is the hall mark of MM (Figure 3). Additionally, MM cells receive crucial signals from the BMM that help them to evade apoptosis in order to maintain their long-term survival. The BM stromal cells (BMSCs) regulate the anti-apoptotic Bcl-2 family proteins by secreting a group of signaling cues. Mcl-1 is regulated through several extracellular signaling molecules including interleukins (IL-3, IL-5, and IL-6) (Wang et al., 1999; Huang et al., 2000; Jourdan et al., 2000); growth factors such as vascular endothelial growth factor (VEGF), epidermal growth factor (EGF) (Leu et al., 2000; Le Gouill et al., 2004); granulocyte macrophage colony stimulating factors (GM-CSF) (Chao et al., 1998); and interferon alpha (INF*-*α) (Jourdan et al., 2000). Combined, these stimuli trigger and modulate multiple signaling pathways including Janus kinase/signal transducer and activator of transcription (JAK/STAT), rat sarcoma/mitogen-activated protein kinase (Ras/MAPK), MEK/extracellular signal-related kinase (ERK) as well as phosphatidylinositol-3 kinase (PI3-K)/Akt (Figure 3).

The cytokine IL-6 is a main survival factor for MM cells (Klein et al., 1995). IL-6 triggers the upregulation of Mcl-1, Bcl-xL, and VEGF *via* stimulation of the JAK/STAT-3 signaling pathway (Puthier et al., 1999; Dankbar et al., 2000). In turn, VEGF promotes IL-6 induction in neighboring BM cells (BMCs) (Dankbar et al., 2000). Furthermore, IL-6 induces survival of MM cells *via* stimulating the Ras/MAPK pathway, which engage in Mcl-1 overexpression (Ogata et al., 1997). Additionally, IFN*-*α induces Mcl-1 in a STAT-3 dependent manner (Jourdan et al., 2000). Furthermore, the tumor necrosis factor (TNF) family including B cell activating factor (BAFF), a proliferation-inducing ligand (APRIL) prevent apoptosis by inducing the expression of Mcl-1 and Bcl-2 (Moreaux et al., 2004). Insulin like growth factor 1 (IGF-1) affects the cell survival, by downregulating pro-apoptotic protein Bim (De Bruyne et al., 2010). The imbalance between Bim and Mcl‐1 expression plays an important role in MM cell survival (Gomez-Bougie et al., 2004). The transcription factors such as B lymphocyte induced maturation protein 1 (Blimp-1), X-box binding protein 1 (XBP-1), and interferon regulatory factor 4 (IRF4) are critical for myeloma cells differentiation and development (Calame et al., 2003). The Blimp-1 downregulates the expression of pro-apoptotic protein Bim (Lin et al., 2007).

Mcl-1 and other anti-apoptotic proteins contain four Bcl-2 homology (BH) domains (BH1-3 domains interact to form a hydrophobic cleft termed “BH3-binding groove”), and a C-terminal tail of hydrophobic transmembrane domain (TM) that permeates into the mitochondrial membrane (Kozopas et al., 1993). Interestingly, compared to the other anti-apoptotic proteins, Mcl-1 has several unique properties including unique binding site, size, half-life, and localization. Mcl-1 has a shallow, relatively inflexible and more electropositive binding site abundant in lysine and histidine residues (Denis et al., 2020). Bcl-2 and Bcl-xL proteins contain 233 amino acids, whereas Mcl-1 protein contains 350 amino acids. This size difference is due to the presence of a large N-terminal domain of four PEST sequences [amino acids sequence extensive in proline (P), glutamic acid (E), serine (S), and threonine (T)] (Kozopas et al., 1993; Thomas et al., 2010), which can target Mcl-1 for degradation through the ubiquitin-proteasome system (UPS) and renders it short half-life (usually less than three hours depending on the cellular conditions) (Rogers et al., 1986; Kozopas et al., 1993; Yang et al., 1995).

In MM, the Mcl-1 gene is the most important and selective of the survival genes (Tiedemann et al., 2012). Gene coding of Mcl-1 is located on chromosome 1q21 region. Approximately 40% of MM cases have shown chromosomal amplification of 1q21, hence increased Mcl-1 expression (Shah et al., 2018; Slomp et al., 2019). Additionally, the gene coding of cytokine interleukin 6 receptor (IL-6R) is located on the same chromosome region (1q21) (Pawlyn and Morgan, 2017). The coding region of Mcl-1 contains three exons and two introns that undergoes alternative splicing to produce mature RNA (mRNA) isoforms. The Mcl-1L (Mcl-1 long) splice variant joins the three exons, has a full length of 350 amino acids and acts as an anti-apoptotic. On the other hand, Mcl-1S (Mcl-1 short) joins only the first and the third exons without the central exon, with length of 271 amino acids, shows increased cytosolic localization and lacks the BH1, BH2 and TM domains but has the BH3 domain which plays a critical pro-apoptotic role (Bae et al., 2000; Bingle et al., 2000). Interestingly, Kim et al. (2009), found a new alternative splicing variant detected in the mitochondrion termed Mcl-1ES (Mcl-1 extra short) with a shorter length of 197 amino acids due to an absence of PEST sequences (Kim et al., 2009). Mcl-1ES forms an interaction with Mcl-1L in order to induce apoptosis (Kim et al., 2009).

The post transcriptional regulation of Mcl-1 is complex and controlled by multiple RNA binding proteins (RPBs) and microRNAs (miRNAs). For example, Mcl-1 has been shown to be downregulated in MM by miR-29b, miR-137, and miR-197 that leads to apoptosis (Zhang et al., 2011; Yang et al., 2015; Cui and Placzek, 2018). Additionally, at the post-translational level, the large N-terminal domain PEST allows for non-proteasomal degradation *via* cleavage (Herrant et al., 2004), proteasomal degradation *via* phosphorylation (Thomas et al., 2010), and ubiquitination (Mojsa et al., 2014), which further impact Mcl-1 expression, stability, localization, and function. Mcl-1 PEST undergoes Caspase cleavage at two different sites, located at Asp127 that produce Mcl-1^1–127^ associated with Mcl-1^128–350^. At Asp158 that produce Mcl-1^1–157^ associated with Mcl-1^158–350^ (Herrant et al., 2004). Interestingly, not all Mcl-1 cleavage fragments revoke anti-apoptotic function. Mcl-1Δ127 fragment has anti-apoptotic function same as Mcl-1 and exists mainly in the cytoplasm and sequester BH3-only or Bak in order to prevent apoptosis (Wang and et al., 2020).

The Mcl-1 phosphorylation plays a critical role in controlling Mcl-1 function as well. Mcl-1 phosphorylation occurs by several protein kinases including; c-Jun N-terminal kinase (JNK) (Inoshita et al., 2002), glycogen synthase kinase 3 (GSK-3) (Maurer et al., 2006; Ding et al., 2007), and extracellular signal-regulated kinase (ERK-1) (Domina et al., 2004; Ding et al., 2008). The phosphorylated Mcl-1 proteins have been reported to result in different functions according to phosphorylation sites (Thomas et al., 2010; Senichkin et al., 2020). Furthermore, a reversible form of post-translational ubiquitination controls several aspects of Mcl-1 including stability and proteasomal degradation and allow for rapid respond to environmental signals in order to change cell state from survival to apoptosis. The Mcl-1 ubiquitin-proteasome system is mediated by five different E3 ubiquitin-ligases including Mcl-1 ubiquitin ligase E3 (Mule) (Zhong et al., 2005), SCF beta-transducin repeats containing protein (SCF^β−TrCP^) (Ding et al., 2007), SCF F-box and WD repeat domain containing 7 (SCF^Fbw7^) (Inuzuka et al., 2011), anaphase-promoting complex/cyclosome (APC/CC^dc20^) (Harley et al., 2010), and tripartite motif containing 17 (Trim17) (Magiera et al., 2013). Furthermore, the ubiquitin-proteasome system contains an additional deubiquitinase called ubiquitin specific peptidase 9, X-linked (USP9X) that removes poly-ubiquitin chains leading to stabilize Mcl-1 and prevent apoptosis (Schwickart et al., 2010). The degree of ubiquitination is also subject to variation based upon the variable phosphorylation of residues of Mcl-1 (Maurer et al., 2006; Ding et al., 2007). Our understanding of Mcl-1 regulations has been greatly expanded by the findings that have developed over the years and provide deep critical insights into exactly how Mcl-1 protein plays such a key role in cellular apoptosis as well as how it can be modulated to provide new options of potential therapeutic approach in MM and other Mcl-1 dependent cancers.

## Development of Selective Mcl-1 Inhibitors

**FIGURE 4:**
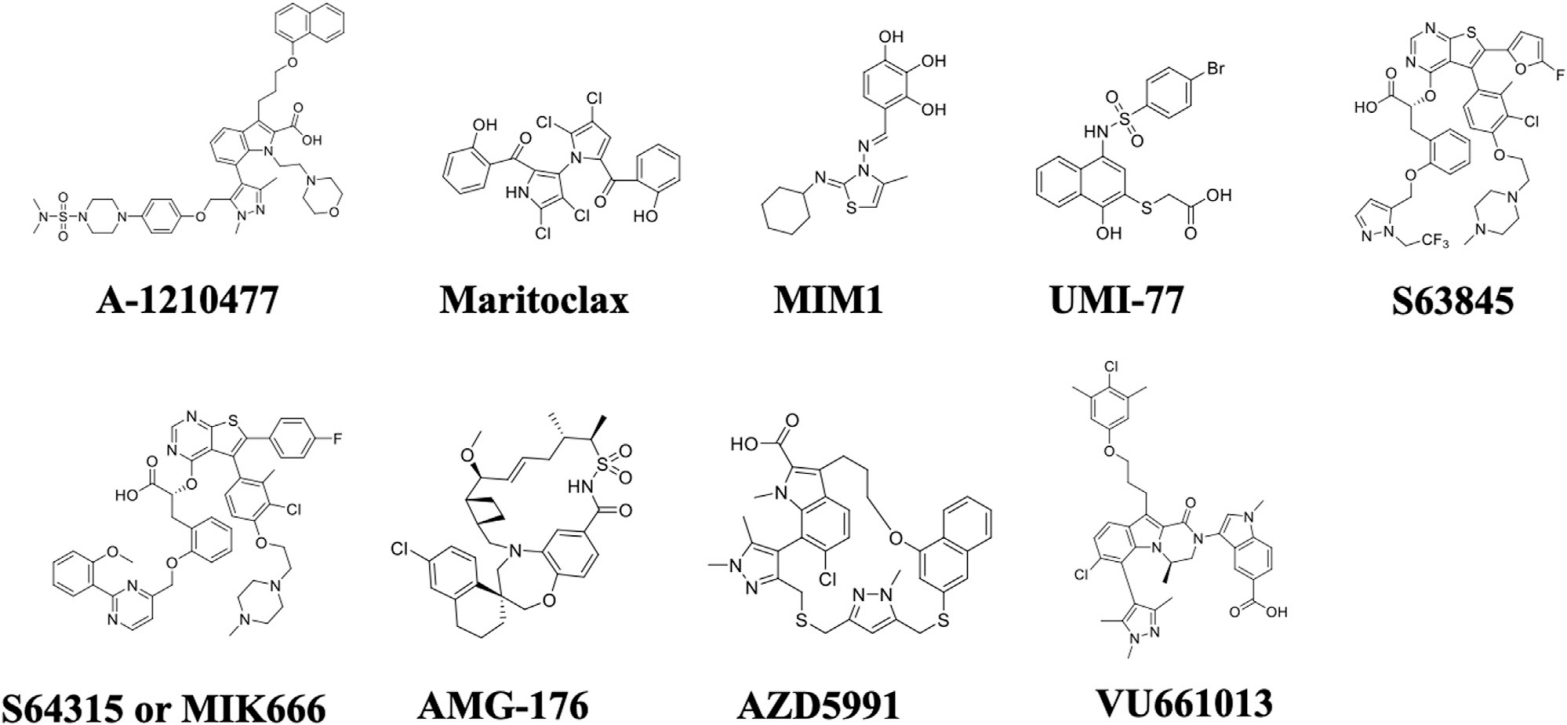
Chemical structures of selective Mcl-1 inhibitors. The most prominent Mcl-1 inhibitors including A-1210477, Maritoclax, MIM1, UMI-77, S63845, S64315/MIK666, AMG-176, AZD5991, and VU661013.

Studies have demonstrated that MM depends on Mcl-1 proteins for survival, prognosis, and chemo resistance. Thus, inhibition of Mcl-1 offers an attractive target and a promising strategy for myeloma treatment. Nonetheless, the targeting of Mcl-1 has been challenging because of its complex regulation. So far, two approaches have been adopted to inhibit Mcl-1, one is direct inhibition and second is indirect targeting. The indirect targeting is a less selective method inhibits other anti-apoptotic proteins, may have more serious side effects. Whereas, direct Mcl-1 inhibitors target the hydrophobic cleft BH3-binding groove of BH3-only proteins interactions domain. Therefore, these inhibitors are very specific to Mcl-1. Here we will review BH3-mimetic inhibitors that selectively bind Mcl-1. The structure of these inhibitors are shown in Figure 4. The current status of development of these agents are summarized in Table 2.

**TABLE 2:** Direct Mcl-1 Inhibitors BH3 Mimetic and semi BH3 Mimetic drugs.

Mcl-1 inhibitor	Company	Affinity	Clinical trial status
A-1210477	Abbive	Ki = 0.45 nM	Preclinical
Maritoclax	Hong-Gang Wang’s group at Pennsylvania State University	IC50 10 μM	Preclinical
MIM1	Cohen and coworkers	Only at very high concentration	Failed *in vivo*
UMI-77	Zaneta Nikolovska-Coleska’s group at University of Michigan	Ki = 490 nM	Preclinical
S63845	Servier and Vernalis	Kd = 0.19 nM	Preclinical
S64315/MIK666	Servier and Vernalis and Novartis	undisclosed	Phase I by Novartis, in R/R lymphoma or R/R MM patients (NCT02992483)
			Phase I by Servier, in AML and MDS patients (NCT02979366)
			Phase I by Servier as a combination of S64315/MIK666 plus Venetoclax in AML patients (NCT03672695)
AMG-176	Amgen	Ki = 0.06 nM	Phase I in R/R MM and R/R AML patients (NCT02675452)
			Phase I as a combination of AMG-176 plus Venetoclax in different R/R hematologic malignancies including AML, NHL, and DLBCL (NCT03797261)
AMG-397	Amgen	undisclosed	Phase I clinical trial is evaluating the safety, tolerability, pharmacokinetics, and efficacy of AMG 397 in MM, AML, DLBCL, and NHL patients (NCT03465540)
AZD5991	AstraZeneca	Ki = 0.2 nM	Phase I as a monotherapy in different R/R hematologic malignancies including NHL, ALL, RS, SLL, T-cell lymphoma, CTCL, CLL, AML/MDS, and MM patients (NCT03218683)
			Phase II is sequential, dose-escalation study of combination AZD5991 plus Venetoclax in R/R AML/MDS patients (NCT03218683)
VU661013	Stephen Fesik’s group at Vanderbilt University	Ki = 0.097 nM	Have partnership with Boehringer Ingelheim Company for clinical trials but no plan disclosed yet. (https://www.boehringer-ingelheim.us/press-release/boehringeringelheim-and-vanderbilt-university-expand-partnership-develop-novel)

### Indole-2-Carboxylic Acids Analog (A-1210477)

This was developed by AbbVie in 2008. A-1210477 induces intrinsic apoptosis pathway by selectively inhibiting Mcl-1 with high binding affinity (Ki = 0.454 nM) (Leverson and et al., 2015). Upon binding, BH3 mimetic A-1210477 results in an accumulation of Mcl-1 protein by preventing its degradation. A-1210477 disrupts the Mcl-1:Bim and Mcl-1:Noxa complexes in order to induce Bax/Bak- dependent MOMP, leading to Cytochrome C release and Caspase activation (Leverson and et al., 2015). The treatment of A-1210477 decreased the association of Mcl-1: Bak complex within an hour, however the complex was totally disrupted after three hours of treatment (Gomez-Bougie et al., 2018). Interestingly, the studies of Mallick et al. (2019) showed that A-1210477 induces rapid apoptosis within 0.5–1 h of treatment, without inducing Noxa (Mallick and et al., 2019). A-1210477 as a monotherapy or in a combination with Navitoclax resulted in death of different cell lines including MM, melanoma, and non-small cell lung cancer cell lines that were found to be Mcl-1 dependent by BH3 profiling or siRNA rescue experiments (Leverson and et al., 2015; Mukherjee and et al., 2018). This finding was reinforced by the efficacy of A-1210477 as a combination with Venetoclax against acute myeloid leukemia (AML) (Fiskus and et al., 2019). A-1210477 inhibited triple negative breast cancer cell line growth activity in vitro which is also considered a Mcl-1 dependent cells type (Campbell and et al., 2018). However, a reference showing A-1210477 induced apoptosis in Bcl-2 dependent cells at higher concentration when compared with Mcl-1 inhibition concentration (Koss et al., 2016). Unfortunately, no in vivo activity was associated with A-1210477, even with the most sensitive cell lines. This was attributed to cell penetration issues and reduced bioavailability due to the high levels of serum protein binding.

### Marinopyrrole A (Maritoclax)

This natural agent was first discovered by Hong-Gang Wangʼs group at Pennsylvania State University in 2012 (Doi et al., 2012). The BH3 mimetic drug Maritoclax induces degradation of Mcl-1 proteins and disrupt Mcl-1: Bim complex. Maritoclax effectively binds the site of the BH3-only proteins p4 binding site and leads to apoptosis. Further, it has been reported that this natural agent is effective against Mcl-1 overexpressing cancer cells (Doi et al., 2012). Blocking BH3 binding site is related with increasing amount of Mcl-1 protein, followed by its ubiquitination and degradation by the E3 ligase (ubiquitin ligase) (Kaleigh and Manabu, 2013). Moreover, the treatment of Maritoclax did not result in Noxa upregulation (Doi et al., 2012). We found that Maritoclax potentiates the apoptotic response of ABT-737 in human melanoma cells (Pandey and et al., 2013).

### Mcl-1 Inhibitor Molecule 1 (MIM1)

Developed in 2012 by Cohen and co-workers, polyphenol compound MIM1 acts as a semi BH3 mimetic which induces Noxa (Cohen et al., 2012). MIM1 seems very similar to BH3 mimetic Mcl-1 inhibitors (Mallick and et al., 2019). This Mcl-1 inhibitor exhibited an ability to induce apoptosis in Mcl-1 dependent cells through upregulation of proapoptotic protein Noxa, which selectively inhibits Mcl-1 with high affinity binding (Smith et al., 2011; Kale et al., 2018; Mallick and et al., 2019). Also, induction of Noxa dissociates Mcl-1:Bim association complex. Unfortunately, MIM1 was only able to induce Bak dependent apoptosis at high concentrations (more than 10 μM). MIM1 failed to induce apoptosis in anti-apoptotic proteins dependent cell lines (Varadarajan et al., 2013).

### UMI-77

Developed in 2013 by Zaneta Nikolovska-Coleskaʼs group at University of Michigan, naphthol derivative UMI-77 is another semi BH3 mimetic Mcl-1 inhibitor with high binding affinity (Ki = 490 nM) (Azmi and et al., 2013; Abulwerdi et al., 2014). In order to induce apoptosis, UMI-77 was found to upregulate pro-apoptotic protein Noxa thereby selectively inhibit Mcl-1 (Mallick and et al., 2019). UMI-77 and Noxa competing for Mcl-1 binding with Bax and Bak proteins ultimately disrupt the Mcl-1: Bak and Mcl-1: Bak complexes, which results in Cytochrome C release and Caspase 3 activation (Abulwerdi et al., 2014). The *in vitro* and *in vivo* preclinical studies demonstrated that UMI-77 potently inhibits tumor growth and induces apoptosis in MM cells (Azmi and et al., 2013), and pancreatic cancer cells lines (Abulwerdi et al., 2014), both of which rely on the Mcl-1 protein as a survival factor (Miyamoto et al., 1999; Schniewind et al., 2004; Ren et al., 2009). In addition to pancreatic cancer cell line BxPC-3 xenograft mouse model and MM animal xenografts, UMI-77 significantly delayed growth activity in breast cancer cell line MDA-MB-468 xenograft mouse model as well (Campbell and et al., 2018).

### S63845

Developed in 2015 by a Servier and Vernalis partnership, atropisomers thienopyrimidine scaffold molecule S63845 is a selective BH3 mimetic Mcl-1 inhibitor that can activates the Bax/Bak dependent mitochondrial apoptotic pathway (Kotschy et al., 2016). S63845 is a selective and potent BH3 mimetic. It binds with high affinity to the BH3-binding groove of Mcl-1 (Kd = 0.19 nM) without any detectable binding to Bcl-2 or Bcl-xL proteins. S63845 showed effective anti-cancer activity in its *in vitro* and *in vivo* preclinical studies (Kotschy et al., 2016). The IV infusion of S63845 once daily for five consecutive days resulted in 100% tumor regression in MM subcutaneous tumor models and lymphoma disseminated mouse model Eμ-Myc (Kotschy et al., 2016; Brennan et al., 2018). The same tumor regression was related with AML as well (Kotschy et al., 2016). This inhibitor had a therapeutic effect without significant weight loss apparent side effects in normal mice tissues (Kotschy et al., 2016). Along with A-1210477 and UMI-77, S63845 also inhibited growth activity of TN breast cancer cell line (Campbell and et al., 2018).

After S63845 proved its eligibility as a selective Mcl-1 antagonist, impressive studies have continued coming up. Recently in 2019, S63845 showed activity both *in vitro* and *in vivo* by killing human T cell acute lymphoblastic leukemia cells (T-ALL) (Li et al., 2019). It was even more potent in inducing apoptosis as a combination therapy with Venetoclax without any appreciable toxicity (Li et al., 2019). In 2020, *in vitro*, *ex vivo,* and *in vivo* preclinical evaluations investigated the combination of S63845 plus Venetoclax. *In vitro* study tested the sensitivities of five MM cell lines to the drug while the *in vivo* study used an aggressive disseminated model of MM. The combined finding came clearly with increasing apoptotic cell death, reduced cell survival as well as delayed tumor growth *in vivo* (Algarín et al., 2020). Furthermore, S63845 was evaluated in a triple combination with Venetoclax plus dexamethasone. Clearly, *in vitro* and *in vivo* studies showed that dexamethasone increased the effectiveness of both S63845 and Venetoclax. Furthermore, *in vitro* studies illustrated that the triple therapy is a stronger synergism than the S63845 plus Venetoclax in resistant MM cell line (MM.1S) (Algarín et al., 2020). In addition, the combination of S63845 and Venetoclax, enhanced the Venetoclax sensitivity and overcome resistance to Venetoclax in human myeloma cell lines (HMCLs) (Wong and Chim, 2020).

Servier and Vernalis and Novartis have created another S-derivative called S64315 or MIK666. S64315/MIK666 is in clinical trial as a single agent in R/R lymphoma or R/R MM (NCT02992483). Furthermore, this molecule being tested in AML and myelodysplastic syndrome (MDS) patients (NCT02979366). Another clinical trial is undergoing by Servier and Vernalis in a combination with Venetoclax in AML patients (NCT03672695).

### AMG-176

Developed in 2016 by Amgen, chirality macrocyclic acylsulfonamide (spiromacrocyclic) AMG-176 is an orally selective Mcl-1 inhibitor with high binding affinity (Ki = 0.06 nM), induces rapid apoptosis in different hematologic malignancies. The treatment of AMG-176 disrupts the interactions of the Mcl-1: Bak complex (Caenepeel et al., 2018; Caenepeel and et al., 2017). Preclinical studies have demonstrated that AMG-176 is non-toxic and efficacious in both MM subcutaneous xenograft models and disseminated models, inhibited 100% tumor growth (Caenepeel et al., 2018). In preclinical studies, AMG-176 has been shown to eradicate CLL cells as a single agent or in a combination with a low dose of Venetoclax (Yi and et al., 2020). Interestingly, AMG-176 was the first selective Mcl-1 inhibitor to be studied in humans. Currently, AMG-176 is in phase I clinical trials *via* IV administrations in patients with R/R MM and patients with R/R AML (NCT02675452). AMG-176 monotherapy has potent anti-myeloma and unique hematologic activity resulting in marked survival improvement. Furthermore, phase I clinical trials have also evaluated AMG-176 as a combination therapy with Venetoclax which presents as an interesting therapy for different R/R hematologic malignancies including AML, diffuse large B cell lymphoma (DLBCL), and Non-Hodgkinʼs lymphoma (NHL) (NCT03797261). Furthermore, as a combination with MEK inhibitor (Trametinib), AMG-176 increased the tumor regression effect in murine models of solid tumor cell lines (Nangia et al., 2018).

Amgen has developed another potent and selective analog AM-8621, nonetheless, this molecule has poor oral bioavailability and short half-life (Caenepeel et al., 2018). Interestingly, MM cells showed sensitivity to AM-8621 as a monotherapy and as a combination therapy with dexamethasone (Caenepeel et al., 2018). Caenepeel et al. (2019) investigated the activities of AMG 176 and AM-8621 in combination with Cytarabine, Doxorubicin, and Decitabine in a preclinical models of AML (Caenepeel and et al., 2019). The other analog AMG-397 is evaluated orally in the clinic. A phase I clinical trial evaluating its safety, tolerability, pharmacokinetics, and efficacy in MM, AML, DLBCL, and NHL patients by administrating AMG-397 in a weekly cycle consisting of two consecutive days of one oral dose followed by five days off at a weekly interval (NCT03465540).

### AZD5991

Developed in 2017 by AstraZeneca, indole-2-carboxylic acids analog AZD5991, is a potent and selective macrocyclic Mcl-1 inhibitor that rapidly activates Caspase proteins, which leads to apoptosis in MM cell lines (GI_50_ = 10 nM) (Hird and et al., 2017; Tron et al., 2018). AZD5991 is a BH3 mimetic with high binding affinity (Ki = 0.2 nM) disrupts the Mcl-1: Bak complex (Hird and et al., 2017; Tron et al., 2018). Most notably, in a number of MM and AML mouse and rat xenograft models, AZD5991 exhibits a potent activity with the preclinical *in vivo* studies showing 100% tumor regression after a single IV dose in both monotherapy and in combination with Venetoclax or Bortezomib (Tron et al., 2018). The preclinical efficacy of AZD5991 is emphasized by the apoptosis and survival improvements in MM models resistant to Venetoclax (Hird and et al., 2017). The remarkable *in vitro* and *in vivo* anti-tumor activities of AZD5991 in both MM and AML models support its consideration as a strong clinical candidate in different Mcl-1 dependent hematologic malignancies. The number of clinical trials are ongoing with AZD5991 as a single agent or in combinations. For example, phase 1 as a monotherapy dose escalation study in several R/R hematologic malignancies including NHL, ALL, Richter syndrome (RS), small lymphocytic lymphoma (SLL), T-cell lymphoma and cutaneous T-cell lymphoma (CTCL) (NCT03218683); phase 1 as a monotherapy in expansion groups of R/R CLL, AML/MDS, and MM patients; and Phase 2 sequential, dose escalation study in combination with Venetoclax in R/R AML/MDS patients (NCT03218683).

### VU661013

Developed in 2017 by Stephen Fesik’s group at Vanderbilt University, indole-2-carboxylic acids analog VU661013 is a potent and selective BH3 mimetic Mcl-1 inhibitor with high binding affinity (Ki = 0.097 nM) (Lee et al., 2017). VU661013 destabilizes the Mcl-1: Bim complex in order to initiate MOMP (Ramsey et al., 2018). VU661013 proved potency in Mcl-1 inhibition in both *in vitro* and *in vivo* studies through its induction of apoptosis in a variety of Mcl-1 dependent tumors. Furthermore, it demonstrated efficacy in combination with Venetoclax in Venetoclax resistant cells, patient derived xenografts, and murine models of AML (Ramsey et al., 2018). The further modifications of this molecule is being made to improve the efficacy and bioavailability. Another analog has been made (compound 42), which bound to Mcl-1 with picomolar affinity (Ki = 70–300 pM) in order to displace Bim (Lee et al., 2019). Compound 42 showed *in vivo* growth inhibition in xenograft models of MM and AML (Lee et al., 2019).

## Conclusion and Future Direction

The anti-apoptotic protein Mcl-1 is critical in survival and drug resistance of several malignancies including MM (Krajewska et al., 1996; Miyamoto et al., 1999; Andersen et al., 2005; Song et al., 2005; Ding et al., 2007; Boisvert-Adamo et al., 2009; Brotin et al., 2010). Last decade or so has seen tremendous development, various Mcl-1 inhibitors have been developed. These new inhibitors may help in overcoming drug resistance and improve treatment of MM and other hematological malignancies where Mcl-1 is an important survival factor. The numerous BH3 mimetic and semi BH3 mimetic drugs have proved their efficacy in preclinical studies. Hopefully, after clinical trial of one of these numerous drugs receive FDA approval. It may open up a new door in the targeted MM therapy that will help to improve medical approaches, and the outcome of MM patients. Although, Mcl-1 inhibitors have good anti-myeloma activity as a monotherapy in hematological cancer models, most development strategies are focused on combination, which can increase the potential of these molecules. These combinations are shown to be especially valuable if the drug consists of a selective Mcl-1 inhibitor plus an existing drug that inhibits other anti-apoptotic proteins including Bcl-2 and Bcl-xL, and/or drug that induce pro-apoptotic proteins expression. Combination therapies such as selective Bcl-2 proteins inhibitors or proteasome inhibitors (Venetoclax or Bortezomib) have been attempted to improve the therapeutic outcome (Caenepeel et al., 2018; Ramsey et al., 2018; Tron et al., 2018; Moujalled et al., 2019). Furthermore, the triple combination therapy plus dexamethasone has shown a good effect and presents as an effective strategy (Algarín et al., 2020). Interestingly, three of the recently developed BH3-mimetic are in clinical trials as a combination therapy with Venetoclax. These combination strategies allow patients to interrupt treatment for long periods of time and show a successful development for this new drug class. Based on this information, nowadays the small molecule BH3 mimetics and semi mimetics compounds represent the most promising approach for the selective inhibition of Mcl-1. This places the priority on the rational design of novel BH3 mimetic drugs that binds extremely tightly and selectively to Mcl-1 for the better outcome of the treatment.
