# Erratum: Mcl-1 Inhibition: Managing Malignancy in Multiple Myeloma

**DOI:** 10.3389/fphar.2021.758130

**Published:** 2021-08-31

**Authors:** Frontiers Production Office

**Affiliations:** Frontiers Media SA, Lausanne, Switzerland

**Keywords:** multiple myeloma, drug resistant, Mcl-1, Bcl-2 homology 3 mimetics, apoptosis

Due to a production error, several author names were incorrectly written. The names of Omar S. Al-Odat, Robert J. Chitren, Weam O. Elbezanti, Sandeep K. Srivastava, Subash C. Jonnalagadda, and Bharat B. Aggarwal had their initials mistakenly omitted.

The publisher apologizes for this mistake.

